# Supplementary material for: Prasugrel inhibits TLR7-driven autoimmunity in systemic lupus erythematosus by acetylating cGAS
Source: Nat Commun. 2026 Mar 18;17:4147. doi: 10.1038/s41467-026-70794-5 (PMC13153160; doi:10.1038/s41467-026-70794-5)
Supplement: Supplementary file 1 — Reporting Summary [file 41467_2026_70794_MOESM1_ESM.pdf]

nature portfolio

Corresponding author(s): Tao Li

Last updated by author(s): Jan 31, 2026

Reporting Summary

Nature Portfolio wishes to improve the reproducibility of the work that we publish. This form provides structure for consistency and transparency in reporting. For further information on Nature Portfolio policies, see our Editorial Policies and the Editorial Policy Checklist.

Statistics

For all statistical analyses, confirm that the following items are present in the figure legend, table legend, main text, or Methods section.

n/a	Confirmed	
☐	☒	The exact sample size (n) for each experimental group/condition, given as a discrete number and unit of measurement
☐	☒	A statement on whether measurements were taken from distinct samples or whether the same sample was measured repeatedly
☐	☒	The statistical test(s) used AND whether they are one- or two-sided<br>*Only common tests should be described solely by name; describe more complex techniques in the Methods section.*
☒	☐	A description of all covariates tested
☐	☒	A description of any assumptions or corrections, such as tests of normality and adjustment for multiple comparisons
☐	☒	A full description of the statistical parameters including central tendency (e.g. means) or other basic estimates (e.g. regression coefficient) AND variation (e.g. standard deviation) or associated estimates of uncertainty (e.g. confidence intervals)
☐	☒	For null hypothesis testing, the test statistic (e.g. F, t, r) with confidence intervals, effect sizes, degrees of freedom and P value noted<br>*Give P values as exact values whenever suitable.*
☒	☐	For Bayesian analysis, information on the choice of priors and Markov chain Monte Carlo settings
☒	☐	For hierarchical and complex designs, identification of the appropriate level for tests and full reporting of outcomes
☐	☒	Estimates of effect sizes (e.g. Cohen's d, Pearson's r), indicating how they were calculated

Our web collection on statistics for biologists contains articles on many of the points above.

Software and code

Policy information about availability of computer code

Data collection	1. Enzyme linked Immunosorbent Assay data and cell viability data were collected using SpectraMax i3 (Molecular Devices).<br>2. qPCR were performed using Applied Biosystems Step OnePlus system (Applied Biosystems).<br>3. Mass Spectrometry of cGAMP were performed on a triple-quadrupole mass spectrometer (Xevo TQ-S, Waters Corp.) equipped with an electrospray ionization source.<br>4. Images of mouse spleens were acquired using Epson Perfection V850 Pro (EPSON).<br>5. Fluorescence images were acquired using a DeltaVision deconvolution microscope (GE healthcare), SLIDEVIEW VS200 slide scanner (Olympus) and ZEISS LSM 900 Confocal Microscopy (ZEISS).<br>6. HE staining images were acquired using NanoZoomer 2.0HT (HAMAMATSU).<br>7. Sequencing data were generated using Illumina NovaSeq X Plus platform (Illumina).<br>8. Calcein-quenching assay and pDCs validation were analysed by Beckman CytoFlex (Beckman).
Data analysis	1. Statistical analysis was performed with GraphPad Prism (v10.1).<br>2. Mass Spectrometry data were analyzed by Analyst Software (v1.6.3).<br>3. Images of fluorescence analysis were performed with OlyVIA (v3.3.24382)<br>4. Fluorescence intensity analysis was performed with ImageJ and volocity (v6.1).<br>5. Images of HE staining analysis were performed by NDP.View2.<br>6. The docking of prasugrel and human cGAS was performed using Discovery Studio 2022.<br>7. Quality control and pre-processing of the RNA-seq data were performed using fastp (v0.23.2).<br>8. Clean reads were mapped to human reference genome GRCh38 using HISAT2 (v2.2.1).<br>9. Gene expression levels were determined using featureCounts (v2.0.1), and were normalized using DESeq2 (v1.38.3).

10. Flow Cytometry data analysis were performed by FlowJo (v10).
The software used for data analysis was described. No special code were used for data analysis.

For manuscripts utilizing custom algorithms or software that are central to the research but not yet described in published literature, software must be made available to editors and reviewers. We strongly encourage code deposition in a community repository (e.g. GitHub). See the Nature Portfolio guidelines for submitting code & software for further information.

Data

Policy information about availability of data

All manuscripts must include a data availability statement. This statement should provide the following information, where applicable:

- Accession codes, unique identifiers, or web links for publicly available datasets
- A description of any restrictions on data availability
- For clinical datasets or third party data, please ensure that the statement adheres to our policy

All RNA-sequencing data reported in this paper have been deposited in the Genome Sequence Archive in National Genomics Data Center, China National Center for Bioinformation under BioProject accession number PRJCA030851 publicly accessible at https://ngdc.cncb.ac.cn/bioproject. We provided the list of molecules with potential cGAS-binding capabilities in Supplementary Table 1. The primer sequences used for qPCR are provided in Supplementary Table 2. The dilutions and catalogue numbers of the antibodies are listed in Supplementary Table 3. Source data are provided with this paper.

Research involving human participants, their data, or biological material

Policy information about studies with human participants or human data. See also policy information about sex, gender (identity/presentation), and sexual orientation and race, ethnicity and racism.

Reporting on sex and gender	This study included both male and female participants. All analyses in this study were not stratified by sex or gender.
Reporting on race, ethnicity, or other socially relevant groupings	The present study is among the Asian population. All analyses in this study were not stratified by race or ethnicity.
Population characteristics	1. The present study is among the Asian population. The inclusion criteria of RA and DM patients were: age between 18 and 75 years old; no psychiatric disorders; no other serious systemic diseases; no complications and tumors. As control group, healthy donors also matched the above inclusion criteria.<br><br>2. SLE patients, all with confirmed the disease at Peking University People's Hospital or the Renji Hospital of Shanghai Jiao tong University School of Medicine, were selected. The age of patients are between 18 and 80 years old. And all SLE patients matched above inclusion criteria, except self-serious systemic diseases.
Recruitment	Healthy donors' samples were from Jiaxing Central Blood Station. Patients were recruited based on their clinical diagnosis at Peking University People's Hospital, including SLE, RA and DM patients, and Renji Hospital of Shanghai Jiao tong University School of Medicine, mainly including SLE patients. The sex distribution of participants was based on sample availability rather than being pre-established. Healthy donors were also matched according to above population characteristics.
Ethics oversight	The study was approved by the ethics committee of Jiaxing Central Blood Station (No. 2024-005), the ethics committee of Peking University People's Hospital (No. 2019PHB089-01) and the Renji Hospital Ethics Committee of Shanghai Jiao tong University School of Medicine (No. 2013-126). It was conducted in compliance with all relevant ethical regulations.

Note that full information on the approval of the study protocol must also be provided in the manuscript.

Field-specific reporting

Please select the one below that is the best fit for your research. If you are not sure, read the appropriate sections before making your selection.

☒ Life sciences ☐ Behavioural & social sciences ☐ Ecological, evolutionary & environmental sciences

For a reference copy of the document with all sections, see nature.com/documents/nr-reporting-summary-flat.pdf

Life sciences study design

All studies must disclose on these points even when the disclosure is negative.

Sample size	Sample size was determined empirically, at least two independent experiments were conducted with different number of littermates. We usually use at least 5 mice per group. Any deviation from the above mentioned sample size are indicated in figure legends. The number of the independent experiments was indicated in each figure legend.
Data exclusions	No data were excluded from analysis.
Replication	All the findings were reliably reproduced in multiple independent experiments. For all experiments, our data represent at least two independent assays that produce similar conclusions. And we also used different assays and readouts to confirm our findings.
Randomization	Samples or cells were randomly allocated into groups in vitro experiments. Mice were randomly assigned to each group in vivo studies.

Blinding	Researchers were blinded during the data collection and analysis where possible.

Reporting for specific materials, systems and methods

We require information from authors about some types of materials, experimental systems and methods used in many studies. Here, indicate whether each material, system or method listed is relevant to your study. If you are not sure if a list item applies to your research, read the appropriate section before selecting a response.

Materials & experimental systems

n/a	Involved in the study
☐	☒ Antibodies
☐	☒ Eukaryotic cell lines
☒	☐ Palaeontology and archaeology
☐	☒ Animals and other organisms
☒	☐ Clinical data
☒	☐ Dual use research of concern
☒	☐ Plants

Methods

n/a	Involved in the study
☒	☐ ChIP-seq
☐	☒ Flow cytometry
☒	☐ MRI-based neuroimaging

Antibodies

Antibodies used

1. For western blotting:

Antibodies against acetylated-Lysine (9441s, 1:1000) cGAS (31659s, 1:1000) and TLR7 (82658s, 1:1000) were obtained from Cell Signaling Technology.

Antibodies against IRF3 (ab68481, 1:1000) and p-IRF3 (ab76493, 1:1000) were from Abcam.

Antibodies against cGAS (1:1000), Ac-cGAS (K384+K394, 1:200) and Ac-cGAS (K414, 1:200) were generated from our laboratory.

Antibodies against beta-actin (66009-1-Ig, 1:5000), GAPDH (10494-1-AP,1:5000) and alpha-tubulin (66031-1-Ig, 1:5000) were purchased from Proteintech Group.

Antibody against TREX1 (611986, 1:1000) was from BD Biosciences.

2. For Immunofluorescence:

Goat Anti-Mouse IgM mu chain (Alexa Fluor® 488) (ab150121, 1:500) was from Abcam.

Goat anti-Mouse IgG (H+L) Highly Cross-Adsorbed Secondary Antibody, (Alexa Fluor™ 647) (A21236, 1:500) was from Invitrogen.

Antibody against G3BP1 (13057-2-AP, 1:1000) was from Proteintech Group.

3. For fluorescence-activated cell sorting (FACS) analysis:

PE anti-mouse CD11c antibody (117307, 1:200) and APC anti-mouse PDCA-1 antibody (127016, 1:200) were from Biolegend.

Validation

All the commercial antibodies have been verified by the manufactures.

1. Acetylated-Lysine (9441s, RRID: AB_331805, Cell Signaling Technology, https://www.cellsignal.cn/products/primary-antibodies/acetylated-lysine-antibody/9441)

2. cGAS (31659s, RRID: AB_2799008, Cell Signaling Technology, https://www.cellsignal.cn/products/primary-antibodies/cgas-d3o8o-rabbit-monoclonal-antibody/31659)

3. IRF3 (ab68481, RRID: AB_11155653, Abcam, https://www.abcam.cn/products/primary-antibodies/irf3-antibody-epr2418y-ab68481.html)

4. p-IRF3 (ab76493, RRID: AB_1523836, Abcam, https://www.abcam.cn/products/primary-antibodies/irf3-phospho-s386-antibody-epr2346 ab76493.html)

5. Beta-actin (66009-1-Ig, RRID: AB_2687938, Proteintech Group, https://www.ptgcn.com/products/Pan-Actin-Antibody-66009-1-Ig.htm)

6. GAPDH (10494-1-AP, RRID: AB_2263076, Proteintech Group, https://www.ptgcn.com/Products/GAPDH-Antibody-10494-1-AP.htm)

7. Alpha-tubulin (66031-1-Ig, RRID: AB_11042766, Proteintech Group, https://www.ptgcn.com/products/tubulin-Alpha-Antibody-66031-1-Ig.htm)

8. TREX1 (611986, RRID: AB_399407, BD Biosciences, https://www.bdbiosciences.com/zh-cn/products/reagents/western-blotting-and-molecular-reagents/western-blot-reagents/purified-mouse-anti-mouse-trex1.611986)

9. Goat Anti-Mouse IgM mu chain (Alexa Fluor® 488) (ab150121, RRID: AB_2801490, Abcam, https://www.abcam.cn/products/secondary-antibodies/goat-mouse-igm-mu-chain-alexa-fluor-488-ab150121.html)

10. Goat anti-Mouse IgG (H+L) Highly Cross-Adsorbed Secondary Antibody, (Alexa Fluor™ 647) (A21236, RRID: AB_2535805, Invitrogen, https://www.thermofisher.cn/cn/zh/antibody/product/Goat-anti-Mouse-IgG-H-L-Highly-Cross-Adsorbed-Secondary-Antibody-Polyclonal/A-21236)

11. G3BP1 (13057-2-AP, RRID: AB_2232034, Proteintech Group, https://www.ptgcn.com/Products/G3BP1-Antibody-13057-2-AP.htm)

12. TLR7 (82658S, RRID: AB_3662102, Cell Signaling Technology, https://www.cellsignal.com/products/primary-antibodies/toll-like-receptor-7-e4j3z-rabbit-monoclonal-antibody/82658)

13. PE anti-mouse CD11c antibody (117307, RRID: AB_313776, Biolegend, https://www.biolegend.com/en-gb/products/pe-cyanine7-anti-mouse-cd11c-antibody-3086?GroupID=BLG11937)

14. APC anti-mouse PDCA-1 antibody (127016, RRID: AB_1967127, Biolegend, https://www.biolegend.com/en-gb/products/apc-anti-mouse-cd317-bst2-pdca-1-antibody-6316?GroupID=BLG7996)

All home-made antibodies (cGAS and Ac-cGAS) were validated in our publication (Dai et al., 2019, Cell, PMID: 30799039).

Eukaryotic cell lines

Policy information about cell lines and Sex and Gender in Research

Cell line source(s)	U937(CRL-1593.2), L929 (CCL-1), H1299 (CRL-5803) were obtained from ATCC. Bone marrow cells, BMDMs and pDCs were generated by 8-12 weeks old wild-type female mice. PBMCs were obtained from 20 healthy donors, 20 RA patients, 20 DM patients and 100 SLE patients.
Authentication	U937, H1299 and L929 were obtained from ATCC and authenticated by this organization with certificates.
Mycoplasma contamination	All cell lines were tested to be mycoplasma free by PCR.
Commonly misidentified lines (See ICLAC register)	No commonly misidentified lines were used.

Animals and other research organisms

Policy information about studies involving animals; ARRIVE guidelines recommended for reporting animal research, and Sex and Gender in Research

Laboratory animals	Trex1+/– of C57BL/6 background were from D. Barnes and T. Lindahl (Cancer Research UK). Wild-type and Trex1–/– mice (3-week-old) were given daily (i.m.) injection of prasugrel (20 mg/kg). Cgas+/– BALB/c mice were generated in our laboratory. Knockout of the Cgas gene in mice was achieved using CRISPR-Cas9 technology. The following sgRNA sequences were used: sgRNA1: 5'-GTGAGGTCTTGCCAGTAGAG-3', sgRNA2: 5'- TGCTCTTCGGAGAGTAGGCC-3'). MRL/MpJ (000486) and MRL/lpr (000485) mice were purchased from The Jackson Laboratory. Cgas+/– MRL/lpr were generated from Cyagen Biosciences (sgRNA1: 5'-TTCGAAGAAAGGCCGCGAAA-3', sgRNA2: 5'-GAAGTGTGTCACCGCCATAG-3', sgRNA3: 5'-ATATATGGCGGGAACGTAGC-3', sgRNA4: 5'-AAGCAGGTTTCGTGTACCTC-3'). Tlr7+/– mice (S-KO-02979) were purchased from Cyagen Biosciences. All mice were maintained under controlled temperature (22 ± 1°C) and humidity (50%-60%) with a 12h light-dark cycle, and housed in a non-specific pathogen-free facility with ad libitum access to food and water. Throughout the study, experimental and control animals were bred and housed separately.
Wild animals	No wild animals were used in this study.
Reporting on sex	Sex was not considered in Trex1+/– and Tlr7+/– of C57BL/6 mice. 8 weeks old females of BALB/c mice were used in the study. 12-16-week-old females of C57BL/6 were used to construct pristane-induced model. 8-16-week-old females of MRL/MpJ and MRL/lpr were used in the study. All animals in the same genotype were randomly assigned to different experimental group.
Field-collected samples	No field collected animals were used.
Ethics oversight	Euthanasia was performed by carbon dioxide inhalation. All animal experiments were performed in accordance with the National Institutes of Health Guide for the Care and Use of Laboratory Animals and with the approval of the Institutional Animal Care and Use Committee of the National Center of Biomedical Analysis (No. IACUC-DWZX-2022-720).

Note that full information on the approval of the study protocol must also be provided in the manuscript.

Plants

Seed stocks

Report on the source of all seed stocks or other plant material used. If applicable, state the seed stock centre and catalogue number. If plant specimens were collected from the field, describe the collection location, date and sampling procedures.

Novel plant genotypes

Describe the methods by which all novel plant genotypes were produced. This includes those generated by transgenic approaches, gene editing, chemical/radiation-based mutagenesis and hybridization. For transgenic lines, describe the transformation method, the number of independent lines analyzed and the generation upon which experiments were performed. For gene-edited lines, describe the editor used, the endogenous sequence targeted for editing, the targeting guide RNA sequence (if applicable) and how the editor was applied.

Authentication

Describe any authentication procedures for each seed stock used or novel genotype generated. Describe any experiments used to assess the effect of a mutation and, where applicable, how potential secondary effects (e.g. second site T-DNA insertions, mosiacism, off-target gene editing) were examined.

Flow Cytometry

Plots

Confirm that:

☒ The axis labels state the marker and fluorochrome used (e.g. CD4-FITC).

☒ The axis scales are clearly visible. Include numbers along axes only for bottom left plot of group (a 'group' is an analysis of identical markers).

☒ All plots are contour plots with outliers or pseudocolor plots.

☒ A numerical value for number of cells or percentage (with statistics) is provided.

Methodology

Sample preparation	In brief, bone marrow cells were harvested and stained with Fixable Viability Dye eFluo 780 in media with 1% serum, and then cells were performed with Mitochondrial Permeability Transition Pore Assay Kit. pDCs were isolated from spleen of mice using plasmacytoid dendritic cell isolation kit (Miltenyi Biotec, 130-107-093). Cells were sorted and analysed on a Beckman CytoFlex.
Instrument	Beckman CytoFlex (Beckman Coulter)
Software	FlowJo software v10
Cell population abundance	Sample purity was greater than 90%.
Gating strategy	From the forward and side scatter plots of cells, the intact cells were gated. In bone marrow cells, the live cells were gated according to the stain within APC-Cy7 channel and then a gate was set in the 488nm laser to indicate the Calcein AM-positive cells. pDCs were gated as CD11c-positive and PDCA-1-positive cells.

☒ Tick this box to confirm that a figure exemplifying the gating strategy is provided in the Supplementary Information.
