# Supplementary material for: Prasugrel inhibits TLR7-driven autoimmunity in systemic lupus erythematosus by acetylating cGAS
Source: Nat Commun. 2026 Mar 18;17:4147. doi: 10.1038/s41467-026-70794-5 (PMC13153160; doi:10.1038/s41467-026-70794-5)
Supplement: Supplementary file 2 — Supplementary Information [file 41467_2026_70794_MOESM2_ESM.pdf]

Prasugrel inhibits TLR7-driven autoimmunity in systemic lupus erythematosus by acetylating cGAS

Zeng-Lin Guo[1,§], Li-Ming Sun[1,§], Shuai Jiang[1,§], Ming Zhao[1,§], Yuhui Li[2], Jinjing Qian[3], Yakai Fu[3], Chunmei Wu[3], Ying Yuan[1,4], Wen Xue[1], Shao-Zhen Jiang[1,5], Sen-Chao Yuan[1], Xucheng Lv[6], Xingxing Yang[6], Lehua Yin[1], Peng-Peng Zhu[1,4], Yu Yu[1], Xin Xu[1], Kai Wang[1], Qiu-Ying Han[1], Zhuoxin Li[1], Zhi-Hui Su[1], Xi-Ping Yu[1], Jiaqi Wu[1], Hong Cai[1], Tian Xia[1], Yuan Chen[1], Xue-Min Zhang[1,4,5], Wei-Hua Li[1], Ai-Ling Li[1,4,5], Tao Zhou[1,4], Zhanguo Li[2], Qiong Fu[3,*], Xinhua He[1,6,*], and Tao Li[1,4,5,*]

Affiliations:

[1]Nanhu Laboratory, National Center of Biomedical Analysis, Beijing, China.

[2]Department of Rheumatology and Immunology, Peking University People’s Hospital, Beijing, China.

[3]Department of Rheumatology, Renji Hospital, Shanghai Jiao Tong University School of Medicine, Shanghai, China.

[4]Institute of Translational Medicine, Zhejiang University, Hangzhou, China.

[5]School of Basic Medical Sciences, Fudan University, Shanghai, China.

[6]Institute of Pharmacology and Toxicology, Beijing, China.

[§]These authors contributed equally: Zeng-Lin Guo, Li-Ming Sun, Shuai Jiang, Ming Zhao.

*Correspondence: fuqiong@renji.com; hexinhua01@126.com; tli@ncba.ac.cn

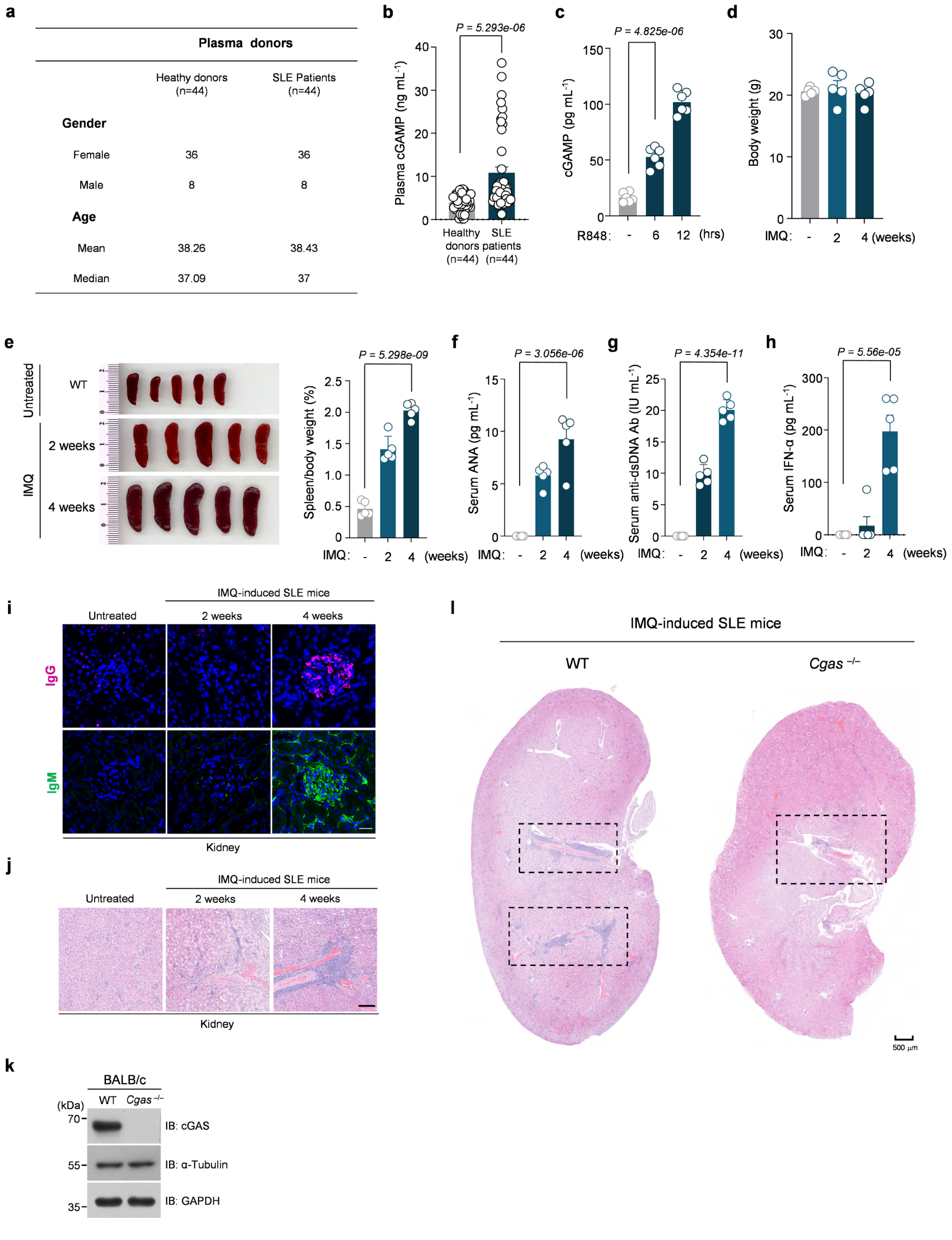
a
Plasma donors
Heathy donors (n=44)
SLE Patients (n=44)
Gender
Female 36 36
Male 8 8
Age
Mean 38.26 38.43
Median 37.09 37
b
P = 5.293e-06
Plasma cGAMP (ng mL-1)
Healthy donors (n=44)
SLE patients (n=44)
c
P = 4.825e-06
cGAMP (pg mL-1)
R848: - 6 12 (hrs)
d
Body weight (g)
IMQ: - 2 4 (weeks)
e
Untreated
WT
IMQ
2 weeks
4 weeks
P = 5.298e-09
Spleen/body weight (%)
IMQ: - 2 4 (weeks)
f
P = 3.056e-06
Serum ANA (pg mL-1)
IMQ: - 2 4 (weeks)
g
P = 4.354e-11
Serum anti-dsDNA Ab (IU mL-1)
IMQ: - 2 4 (weeks)
h
P = 5.56e-05
Serum IFN-α (pg mL-1)
IMQ: - 2 4 (weeks)
i
IMQ-induced SLE mice
Untreated
2 weeks
4 weeks
IgG
IgM
Kidney
j
IMQ-induced SLE mice
Untreated
2 weeks
4 weeks
Kidney
k
BALB/c
WT
Cgas −/−
(kDa)
70
55
35
IB: cGAS
IB: α-Tubulin
IB: GAPDH
l
IMQ-induced SLE mice
WT
Cgas −/−
500 μm

Supplementary Fig.1 cGAS mediates the autoimmunity in TLR7-driven systemic lupus erythematosus.

a,**b**, Table of information of plasma donors (**a**), and plasma cGAMP concentrations of healthy donors and SLE patients (n = 44) (**b**). **c**, Analysis of cGAMP in bone marrow cells at the indicated times after R848 treatment. Data are presented as the means ± SEM (n = 6 independent biological replicates). **d-j**, BALB/c mice (n = 5, per group) were treated with IMQ at indicated times. Body weight of mice was monitored (**d**). The images of spleens of mice and the ratio of spleen to body weight (**e**). ELISA detection of serum anti-nuclear antibodies (**f**), anti-dsDNA antibodies (**g**) and IFN-α concentrations (**h**). Data are presented as the means ± SEM (n = 5 independent biological replicates). Immunofluorescent staining of IgG (purple) and IgM (green) in kidneys from mice (**i**) (n=5 per group). Scale bar, 20 μm. HE-stained section of kidneys from the mice (**j**) (n=5 per group). Scale bar, 200 μm. Representative images are shown. **k**, Immunoblot analysis of WT and *Cgas*$^{-/-}$ bone marrow cells from BALB/c mice with indicated antibodies. **l**, HE-stained of kidney from WT and *Cgas*$^{-/-}$ BALB/c female mice treated with IMQ for 4 weeks (n = 5 per group). Scale bar, 500 μm. Representative images are shown. Statistical significance was determined using two-tailed student's unpaired t-test (**b**); one-way ANOVA with Tukey's test (**c**, **e-h**). P values are shown with figures. Source data are provided as a Source Data file.

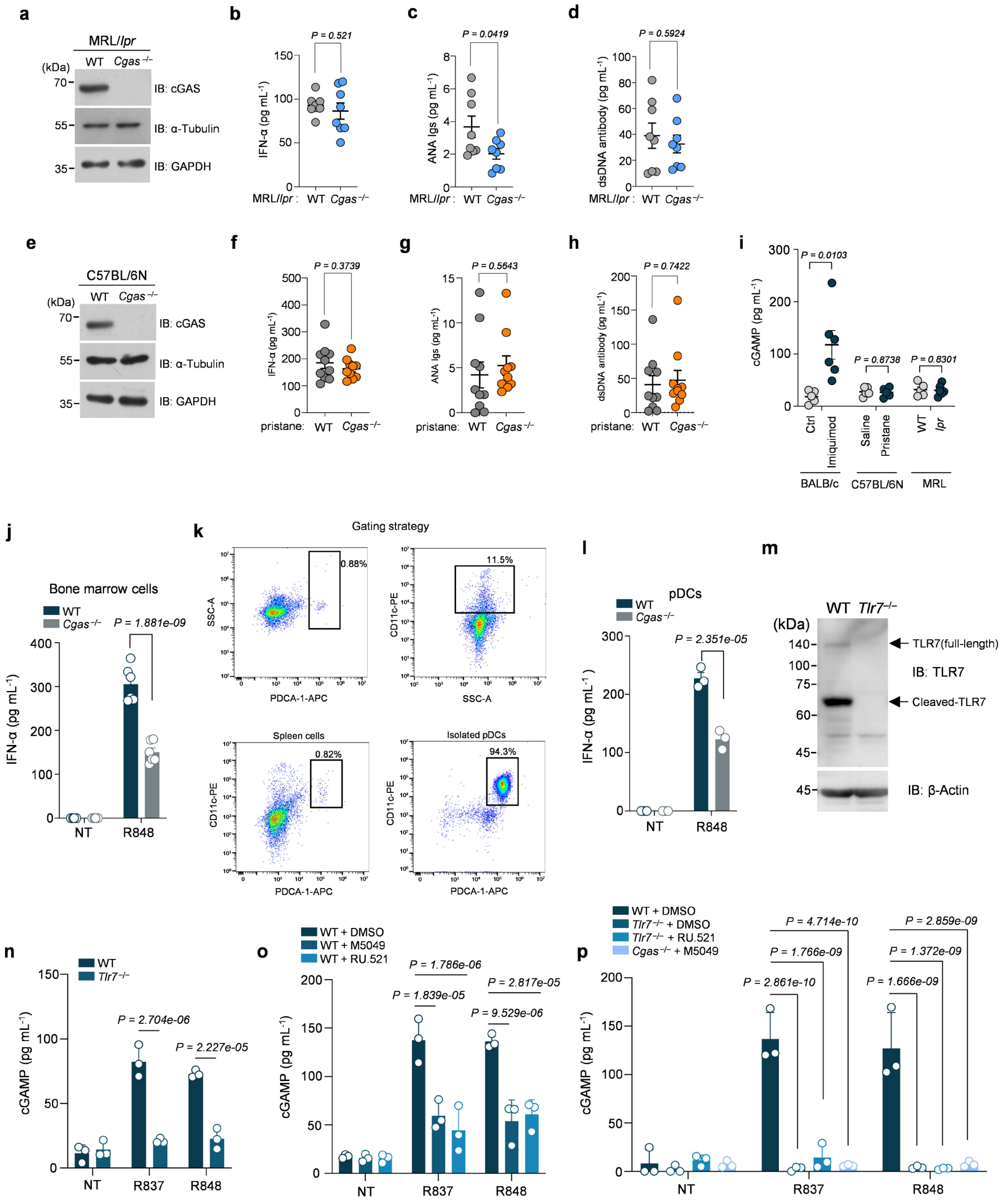

a
MRL/lpr
WT Cgas−/−
(kDa)
70
55
35
IB: cGAS
IB: α-Tubulin
IB: GAPDH
b
P = 0.521
IFN-α (pg mL−1)
MRL/lpr : WT Cgas−/−
c
P = 0.0419
ANA Igs (pg mL−1)
MRL/lpr : WT Cgas−/−
d
P = 0.5924
dsDNA antibody (pg mL−1)
MRL/lpr : WT Cgas−/−
e
C57BL/6N
WT Cgas−/−
(kDa)
IB: cGAS
IB: α-Tubulin
IB: GAPDH
f
P = 0.3739
IFN-α (pg mL−1)
pristane: WT Cgas−/−
g
P = 0.5643
ANA Igs (pg mL−1)
pristane: WT Cgas−/−
h
P = 0.7422
dsDNA antibody (pg mL−1)
pristane: WT Cgas−/−
i
P = 0.0103
P = 0.8738
P = 0.8301
cGAMP (pg mL−1)
Ctrl
Imiquimod
Saline
Pristane
WT
lpr
BALB/c
C57BL/6N
MRL
j
Bone marrow cells
WT
Cgas−/−
P = 1.881e-09
IFN-α (pg mL−1)
NT
R848
k
Gating strategy
0.88%
SSC-A
PDCA-1-APC
11.5%
CD11c-PE
SSC-A
Spleen cells
0.82%
CD11c-PE
PDCA-1-APC
Isolated pDCs
94.3%
CD11c-PE
PDCA-1-APC
l
pDCs
WT
Cgas−/−
P = 2.351e-05
IFN-α (pg mL−1)
NT
R848
m
WT Tlr7−/−
(kDa)
140
100
75
60
45
45
TLR7(full-length)
IB: TLR7
Cleaved-TLR7
IB: β-Actin
n
WT
Tlr7−/−
P = 2.704e-06
P = 2.227e-05
cGAMP (pg mL−1)
NT
R837
R848
o
WT + DMSO
WT + M5049
WT + RU.521
P = 1.786e-06
P = 1.839e-05
P = 2.817e-05
P = 9.529e-06
cGAMP (pg mL−1)
NT
R837
R848
p
WT + DMSO
Tlr7−/− + DMSO
Tlr7−/− + RU.521
Cgas−/− + M5049
P = 4.714e-10
P = 1.766e-09
P = 2.861e-10
P = 2.859e-09
P = 1.372e-09
P = 1.666e-09
cGAMP (pg mL−1)
NT
R837
R848

Supplementary Fig.2 cGAS is involved in TLR7 signaling.

a, Immunoblot analysis of WT and *Cgas*$^{-/-}$ bone marrow cells from MRL/*lpr* mice with indicated antibodies. **b**-**d**, ELISA detection of serum IFN-α concentrations, anti-nuclear antibodies and anti-dsDNA antibodies of WT and *Cgas*$^{-/-}$ MRL/*lpr* mice (n = 8). Data are presented as the means ± SEM. **e**, Immunoblot analysis of WT and *Cgas*$^{-/-}$ bone marrow cells from C57BL/6N mice with indicated antibodies. **f**-**h**, ELISA detection of serum IFN-α concentrations, anti-nuclear antibodies and anti-dsDNA antibodies of WT and *Cgas*$^{-/-}$ C57BL/6N mice (n = 10). Data are presented as the means ± SEM. **i**, Analysis of cGAMP in serum of IMQ-induced mice and Pristane-induced mice for one week after the initial treatments. 8-10-week-old MRL/*lpr* mice were used to measure the serum cGAMP levels. Data are presented as the means ± SEM (n = 5-6). **j**, Bone marrow cells from WT and *Cgas*$^{-/-}$ mice were treated with R848 and the IFN-α concentrations were measured by ELISA. Data are presented as the means ± SEM (n = 6). **k**, Gating strategy to sort pDCs and FACS analysis of the percentage of pDC isolated from spleen of mice. **l**, pDCs from WT and *Cgas*$^{-/-}$ mice were treated with R848 and the IFN-α concentrations were measured by ELISA. **m**, Immunoblot analysis of WT and *Tlr7*$^{-/-}$ bone marrow cells from mice with indicated antibodies. **n**, Bone marrow cells from WT and *Tlr7*$^{-/-}$ mice were treated with R837 or R848 and analysis of cGAMP concentrations were performed. **o**, Analysis of cGAMP in bone marrow cells pretreated as indicated and then treated with R837 or R848. **p**, Analysis of cGAMP in WT, *Tlr7*$^{-/-}$ and *Cgas*$^{-/-}$ bone marrow cells pretreated as indicated and then treated with R837 or R848. NT, non-treated. Data are presented as the means ± SEM from three independent experiments (**l**, **n**-**p**). Statistical significance was determined using two-tailed student's unpaired *t*-test (**c**-**i**); two-way ANOVA with Tukey's test (**j**, **l**, **n**-**p**). *P* values are shown with figures. Source data are provided as a Source Data file.

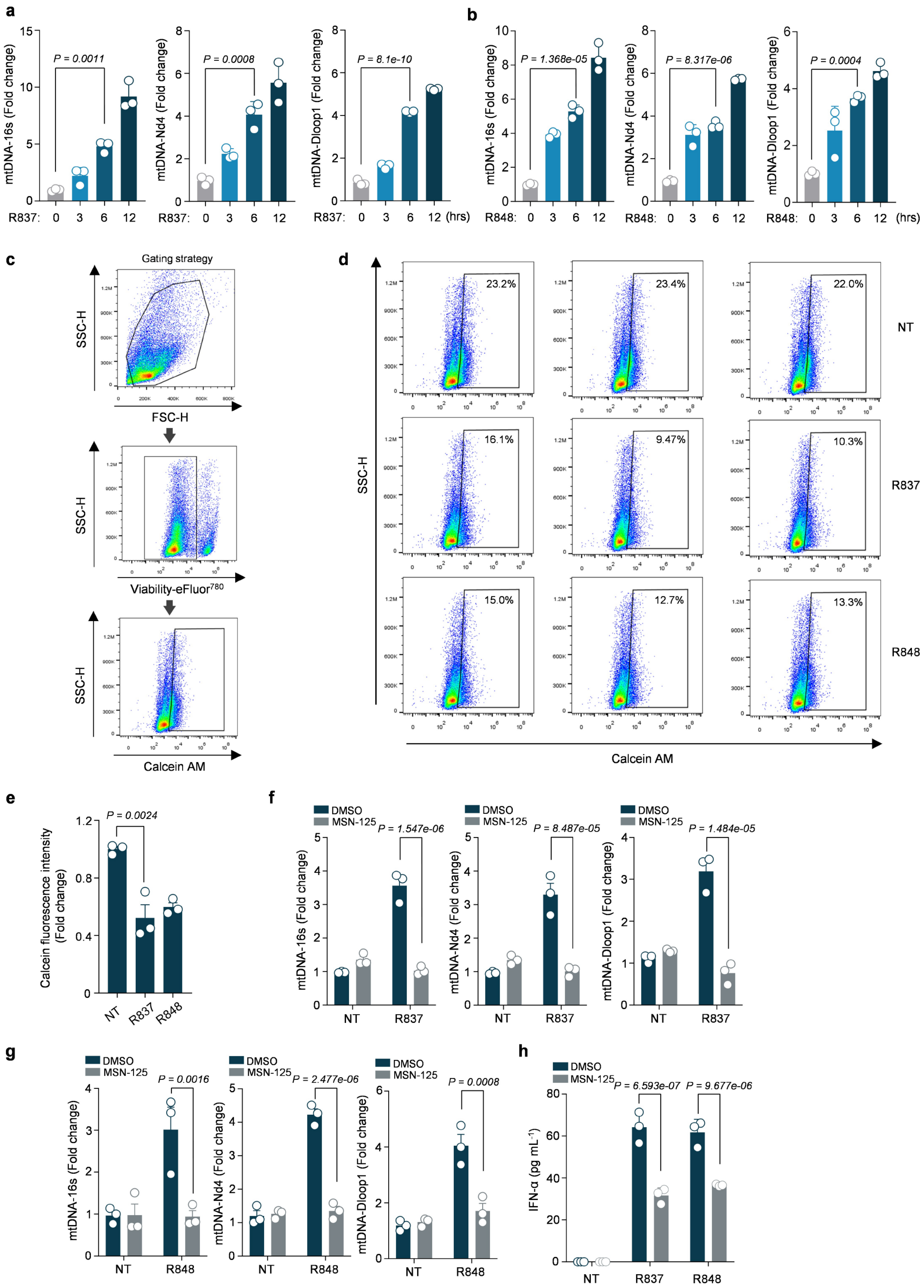
a
mtDNA-16s (Fold change)
P = 0.0011
R837: 0 3 6 12
mtDNA-Nd4 (Fold change)
P = 0.0008
R837: 0 3 6 12
mtDNA-Dloop1 (Fold change)
P = 8.1e-10
R837: 0 3 6 12 (hrs)
b
mtDNA-16s (Fold change)
P = 1.368e-05
R848: 0 3 6 12
mtDNA-Nd4 (Fold change)
P = 8.317e-06
R848: 0 3 6 12
mtDNA-Dloop1 (Fold change)
P = 0.0004
R848: 0 3 6 12 (hrs)
c
Gating strategy
SSC-H
FSC-H
Viability-eFluor780
Calcein AM
d
SSC-H
23.2%
23.4%
22.0%
NT
16.1%
9.47%
10.3%
R837
15.0%
12.7%
13.3%
R848
Calcein AM
e
Calcein fluorescence intensity (Fold change)
P = 0.0024
NT
R837
R848
f
DMSO
MSN-125
P = 1.547e-06
mtDNA-16s (Fold change)
P = 8.487e-05
mtDNA-Nd4 (Fold change)
P = 1.484e-05
mtDNA-Dloop1 (Fold change)
NT
R837
g
DMSO
MSN-125
P = 0.0016
mtDNA-16s (Fold change)
P = 2.477e-06
mtDNA-Nd4 (Fold change)
P = 0.0008
mtDNA-Dloop1 (Fold change)
NT
R848
h
DMSO
MSN-125
P = 6.593e-07
P = 9.677e-06
IFN-α (pg mL-1)
NT
R837
R848

Supplementary Fig.3 TLR7 activation-triggered cytosolic mtDNA release activates cGAS

a,**b**, qPCR analysis of cytosolic mtDNA (*16s*, *Nd4* and *Dloop1*) levels in bone marrow cells treated with R837 or R848 for indicated times. Data are presented as the means ± SEM from three independent experiments. **c**, Gating strategy to determine the calcein fluorescence. **d**, Calcein fluorescence analysis in bone marrow cells treated with R837 or R848 from three independent experiments. **e**, Relative calcein fluorescence in bone marrow cells treated with R837 or R848. Data are presented as the means ± SEM from three independent experiments. **f**-**g**, Bone marrow cells incubated with DMSO or MSN-125 (10 μM) for 3 h, followed by treated with R837 or R848. qPCR analysis of cytosolic mtDNA levels (**f**,**g**) or ELISA analysis of IFN-α secretion as indicated (**h**). Data are presented as the means ± SEM from three independent experiments. NT, non-treated. Statistical significance was determined using one-way ANOVA with Tukey's test (**a**, **b**, **e**) and two-way ANOVA with Tukey's test (**f**-**h**). *P* values are shown with figures. Source data are provided as a Source Data file.

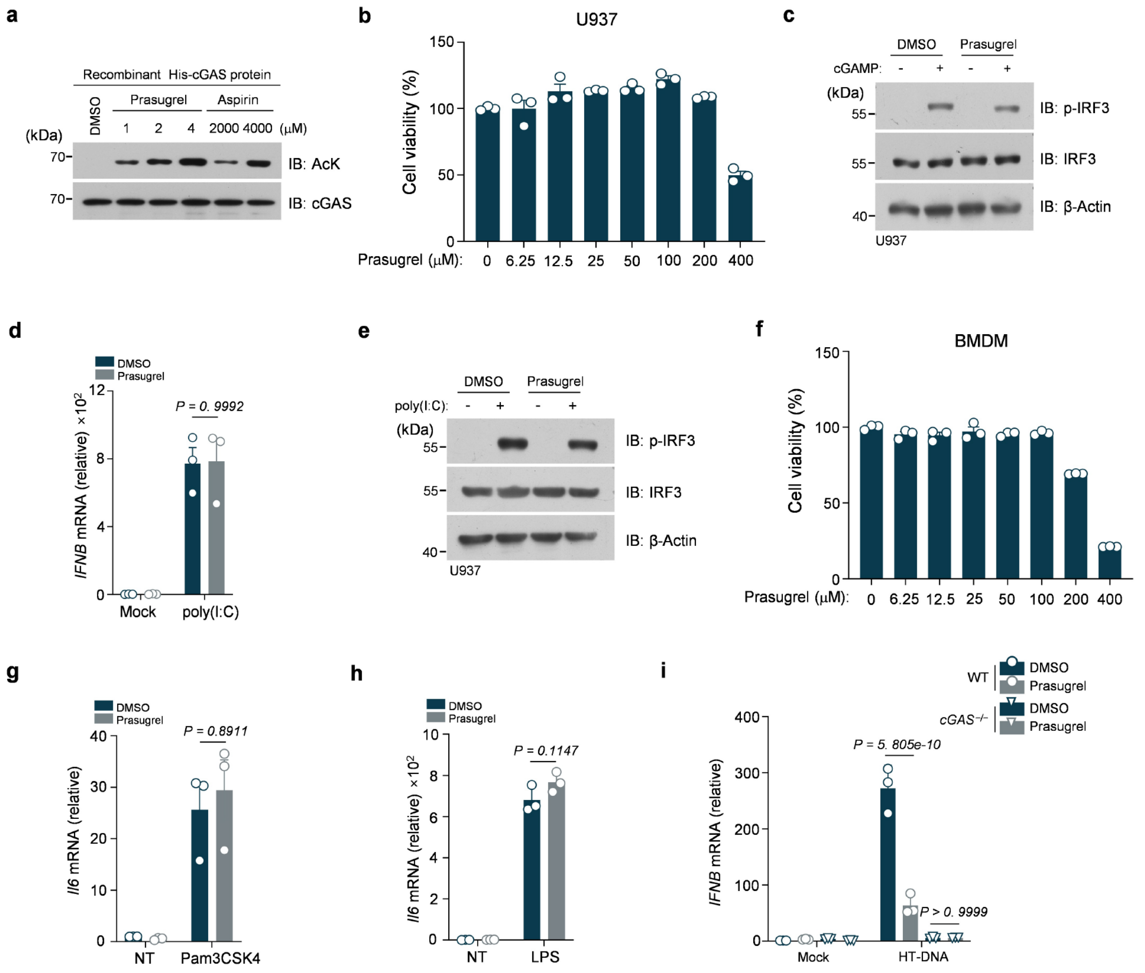

Supplementary Fig.4 Prasugrel specifically inhibits DNA-mediated IFN production.

a, Immunoblot analysis of cGAS acetylation by prasugrel or aspirin using anti-pan-acetyl-lysine antibodies (AcK). Representative of three independent experiments. **b**, U937 cells (PMA-differentiated, hereinafter the same unless otherwise indicated) were incubated with prasugrel at indicated concentrations for 24 h. Cell viabilities were assessed using MTS assay. Data are presented as the means ± SEM from three independent experiments. **c**-**e**, U937 cells were pretreated with DMSO or prasugrel for 24 h, followed by the cGAMP (1 μg/mL) treatment (**c**) or poly(I:C) transfection (1 μg/mL) (**d**,**e**). Immunoblot analysis of indicated proteins (**c**,**e**), representative of three independent experiments. qPCR analysis of *IFNB* mRNA expression (**d**). Data are presented as the means ± SEM from three independent experiments. **f**, BMDMs were incubated with prasugrel at indicated concentrations for 24 h. Cell viabilities were assessed using MTS assay. Data are presented as the means ± SEM from three independent experiments. **g**,**h**, U937 cells were pretreated with DMSO or prasugrel for 24 h, followed by Pam3CSK4 (1 μg/mL) or LPS (0.5 μg/mL) challenges. qPCR analysis of *Il6* mRNA expression. Data are presented as the means ± SEM from three independent experiments. **i**, qPCR analysis of *IFNB1* mRNA expression in WT and cGAS$^{-/-}$ cells transfected with HT-DNA (2 μg/mL) following the pretreatment with DMSO or prasugrel for 24 h. Data are presented as the means ± SEM from three independent experiments. Statistical significance was determined using two-way ANOVA with Tukey's test (**d**, **g**-**i**). *P* values are shown with figures. Source data are provided as a Source Data file.

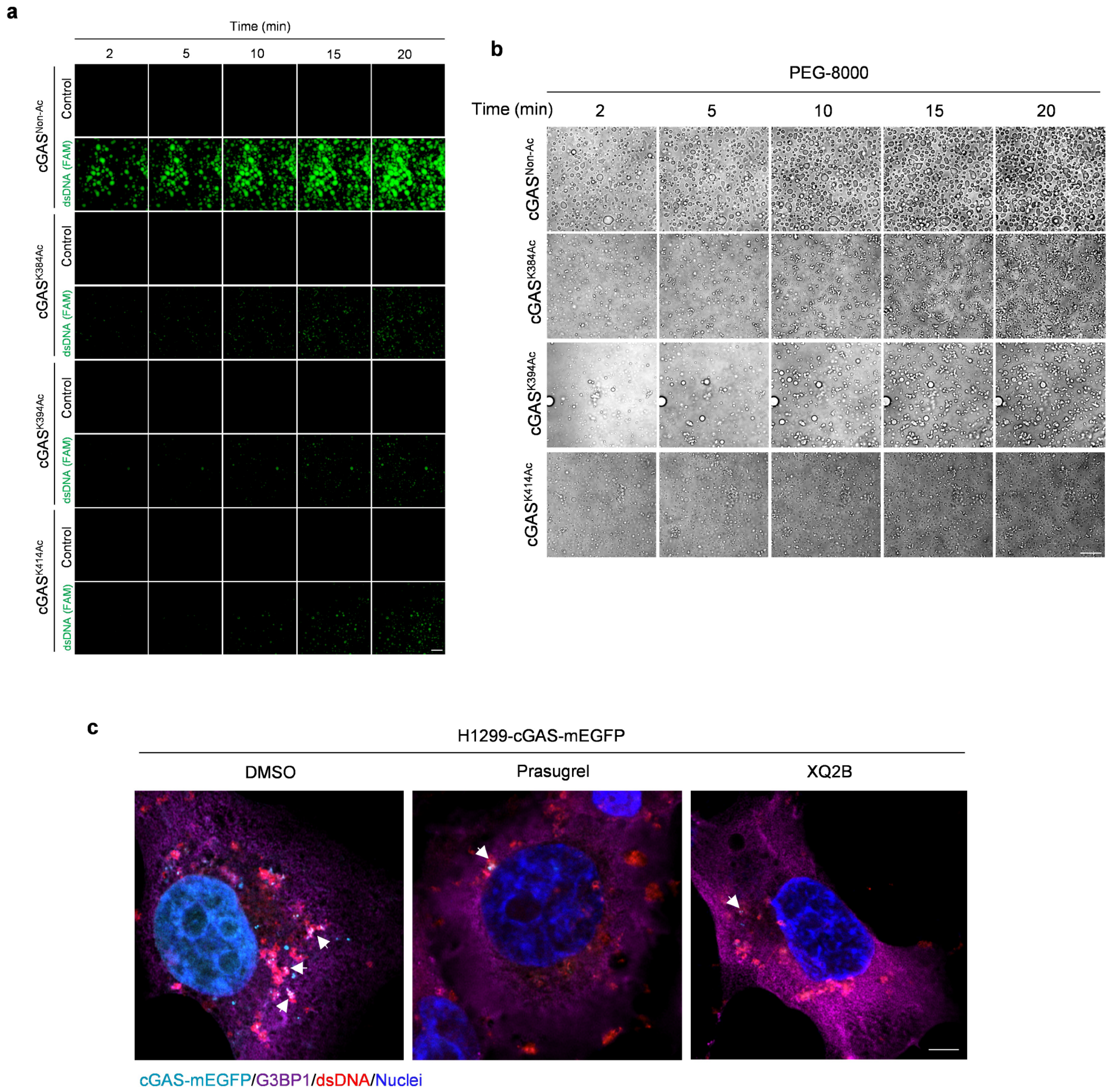

Supplementary Fig.5 Acetylation disrupts DNA-induced phase condensation of cGAS.

a, Recombinant cGAS^{Non-Ac}, cGAS^{K384Ac}, cGAS^{K394Ac} and cGAS^{K414Ac} proteins were incubated with or without (control) FAM-labeled dsDNA. The phase condensations of cGAS proteins were observed with fluorescence microscope. Representative of three independent experiments. Scale bar, 25 μm. **b**, Bright-field microscope images of indicated groups with PEG-8000 treatment. Representative of three independent experiments. Scale bar, 25 μm. **c**, H1299-cGAS-mEGFP were pretreated with DMSO, prasugrel or XQ2B, followed by the Cy3-DNA transfection (4 μg/mL). Immunofluorescence analysis of G3BP1 (purple), cGAS (cyan) and Cy3-DNA (red) in H1299-cGAS-mEGFP cells, showing a colocalization of cGAS with DNA. Hoechst (blue) stained the nuclei. Representative of three independent experiments. Scale bar, 5 μm.

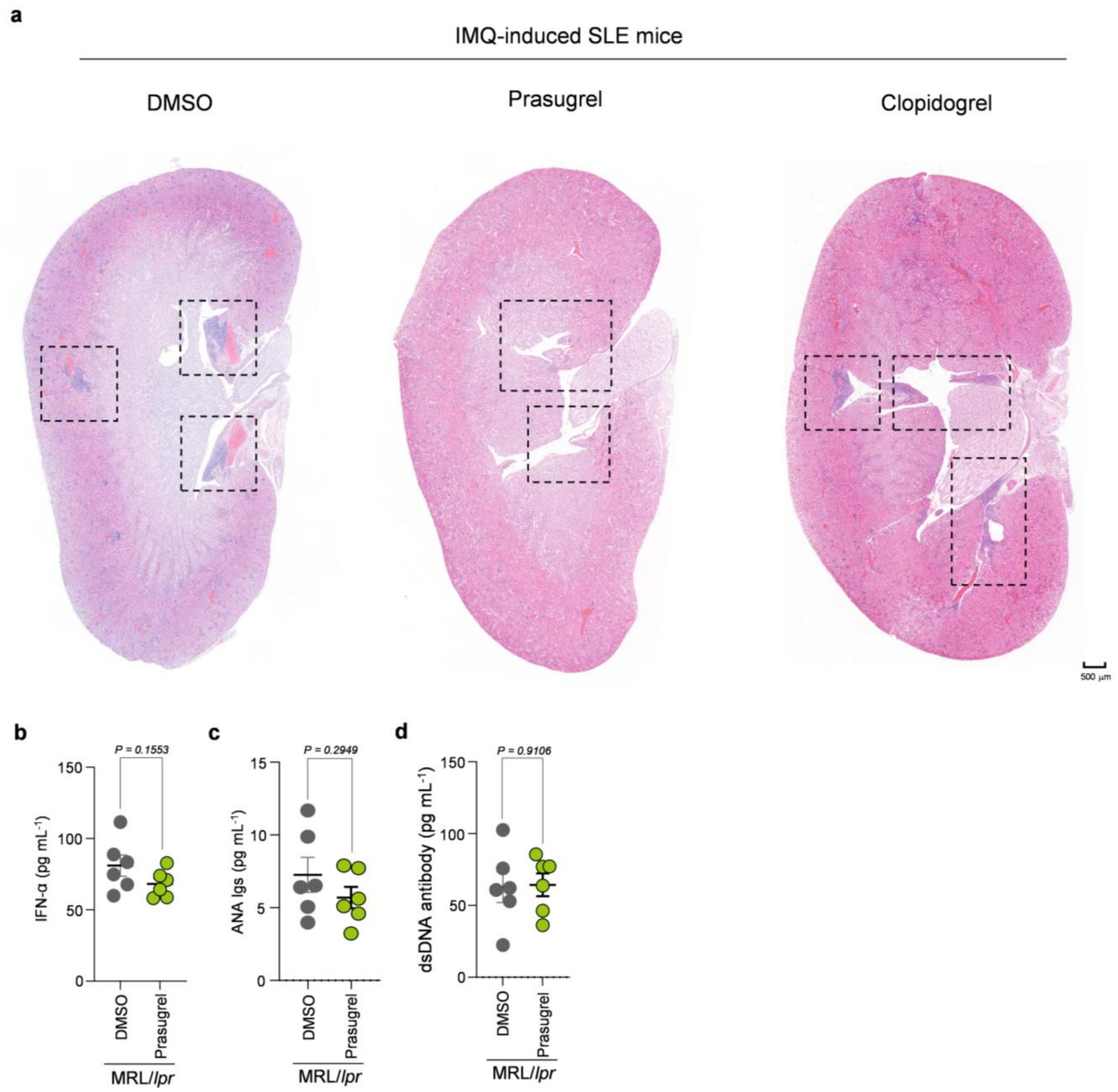

Supplementary Fig.6 Prasugrel ameliorates autoimmunity in SLE mice.

a, HE-stained of kidneys from the BALB/c female mice treated with IMQ for 4 weeks in indicate treatment (n = 8 per group). Representative images are shown. Scale bar, 500 μm. **b-d**, ELISA detection of serum IFN-α concentrations, anti-nuclear antibodies and anti-dsDNA antibodies of MRL/*lpr* mice with DMSO or prasugrel treatment. Data are presented as the means ± SEM (n = 6 independent biological replicates). Statistical significance was determined using two-tailed student's unpaired *t*-test (**b-d**). *P* values are shown with figures. Source data are provided as a Source Data file.

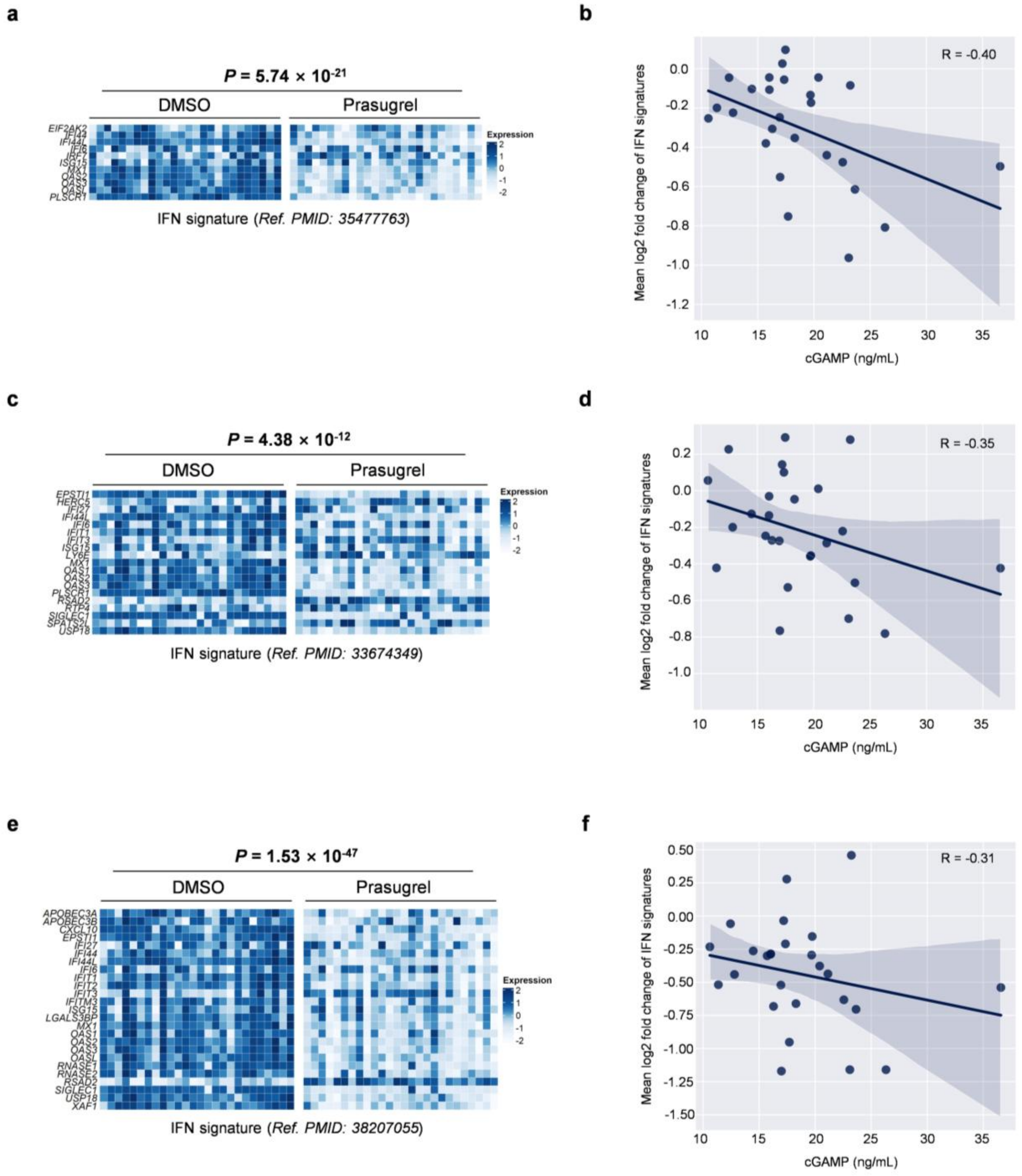

Supplementary Fig.7 Prasugrel exhibits potential therapeutic effects in SLE patient cells.

a-**f**, Heatmaps showing relative expression of genes of IFN signatures in SLE patient PBMCs treated with DMSO or prasugrel (n = 26) (**a**,**c**,**e**). Association between cGAMP levels and mean log2-transformed fold change of expression levels of IFN signature genes quantified using Pearson's correlation coefficient (**b**,**d**,**f**). The solid line indicates the linear regression fit. The error bands represent the 95% confidence intervals. Statistical significance was determined using two-tailed student's paired *t*-test (**a**,**c**,**e**). *P* values are shown with figures. IFN signature gene lists (**a**, **b**, *Ref. PMID: 35477763*; **c**,**d**, *Ref. PMID: 33674349*; **e**,**f**, *Ref. PMID: 38207055*). Source data are provided as a Source Data file.

Supplementary table 1. List of FDA-approved small molecules predicted to bind cGAS by molecular docking.			
NO.	Name	Structure	CDOCKER interaction energy
1	Laninamivir octanoate	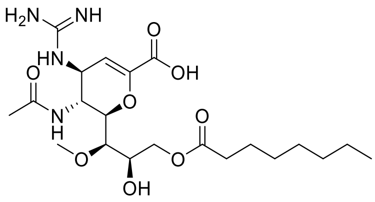	73.11
2	Cefathiamidine	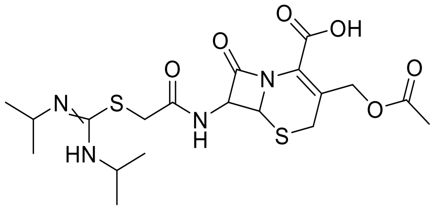	64.49
3	Cefotaxime	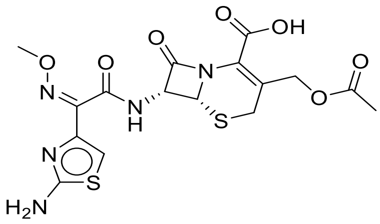	63.43
4	Bisacodyl	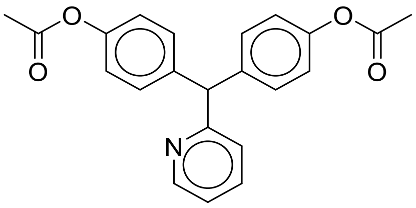	59.91
5	Cephapirin Sodium	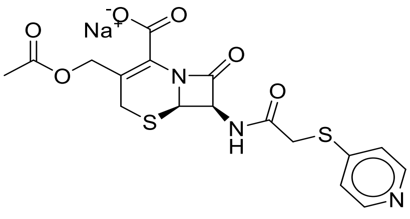	57.86
6	Racecadotril	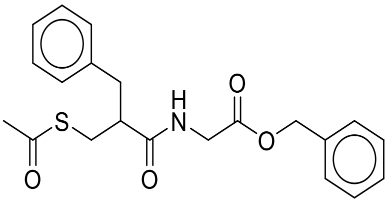	55.43

7	Acebutolol hydrochloride	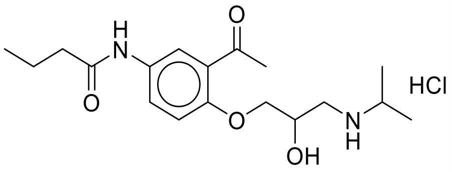	54.65
8	Prasugrel	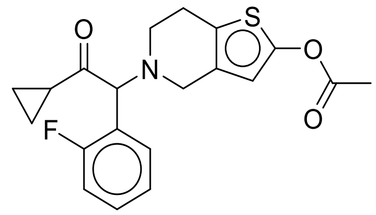	53.55
9	Cephalothin sodium	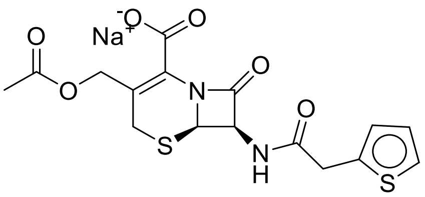	53.48
10	Idarubicin hydrochloride	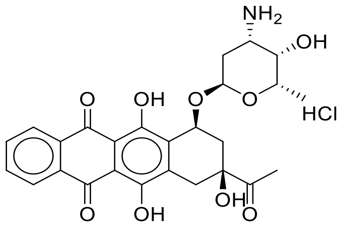	53.47
11	Diltiazem hydrochloride	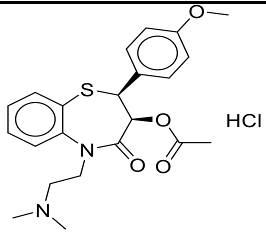	53.46
12	Apremilast	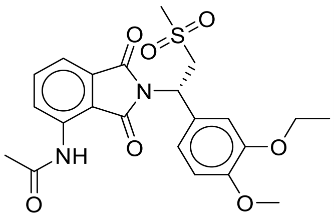	53.3
13	Famciclovir	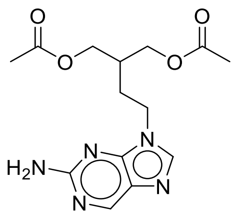	53.27
14	Roxatidine Acetate hydrochloride	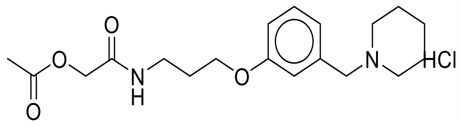	53.14

15	Ketoconazole	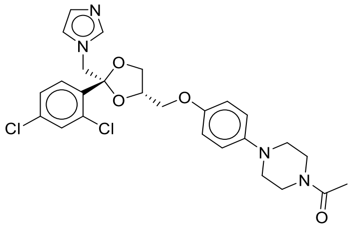	53.11
16	Zanamivir	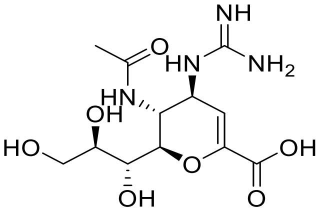	52.68
17	Oseltamivir phosphate	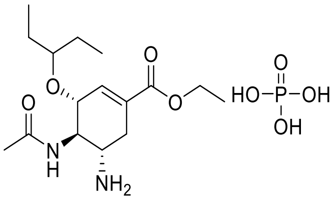	52.55
18	5-Acetylsalicylic acid	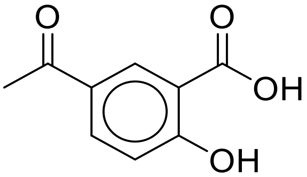	52.47
19	Warfarin sodium	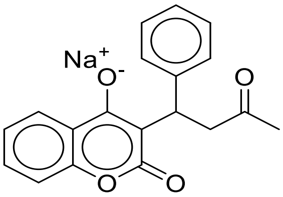	52.4
20	N-Acetylneuraminic acid	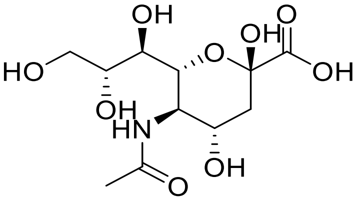	52.339
21	Acenocoumarol	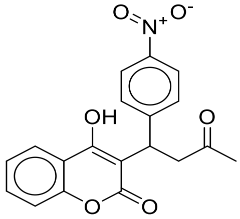	52.332
22	Gestonorone Capronate	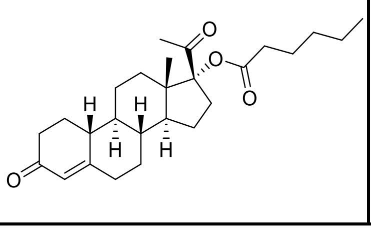	52.29

23	Rociletinib	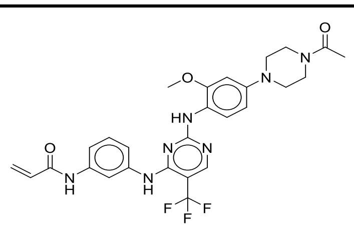	51.98
24	Iloperidone	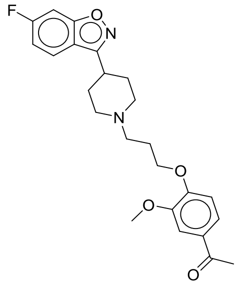	51.75
25	Cefuroxime axetil	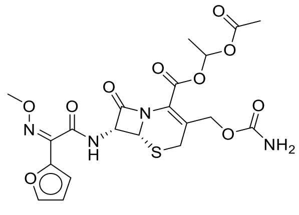	51.67
26	Piperacetazine	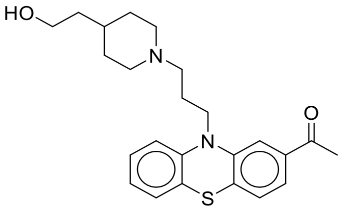	51.38
27	Abiraterone Acetate	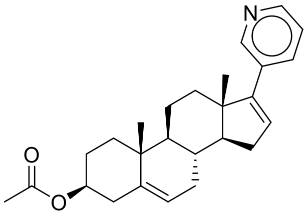	51.37
28	Alacepril	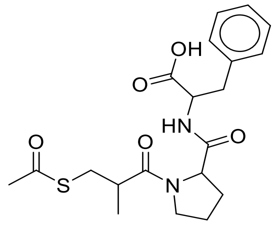	51.27
29	Armillarisin A	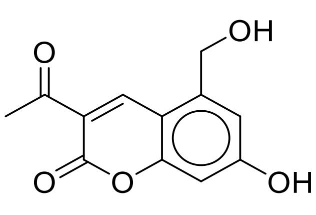	51.055
30	Peramivir Trihydrate	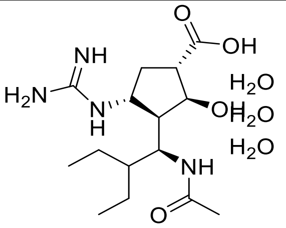	51.05

31	Daunorubicin hydrochloride	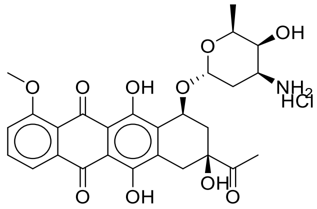	51.038
32	Flurbiprofen Axetil	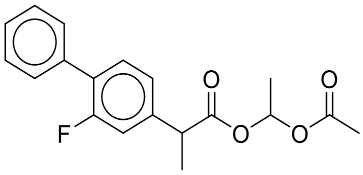	51.031
33	Ioxitalamic Acid	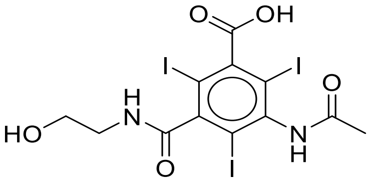	50.96
34	Oseltamivir acid	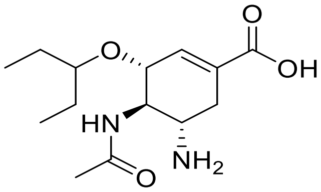	49.37
35	Auranofin	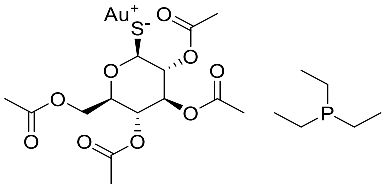	49.29
36	Diacerein	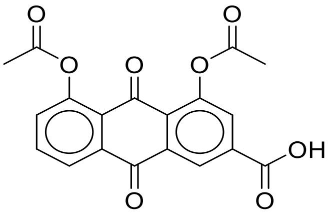	48.88
37	Hydroxyprogesterone caproate	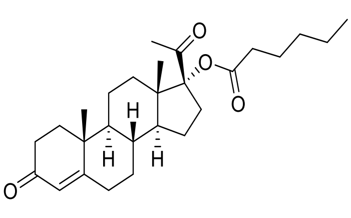	48.59
38	Usnic Acid	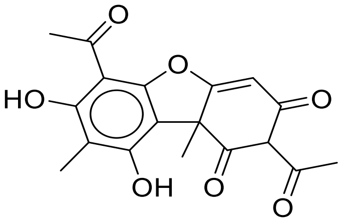	48.58

39	Medroxyprogesterone Acetate	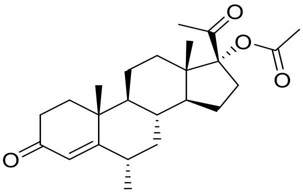	48.45
40	Linezolid	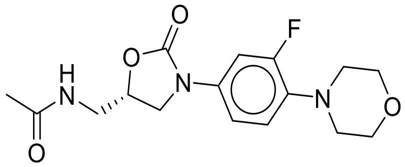	48.43
41	D-α-Tocopherol acetate	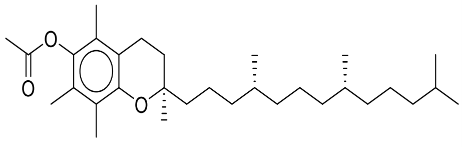	48.02
42	Teprenone	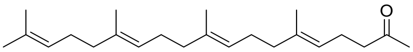	47.93
43	N-Acetylprocainamide	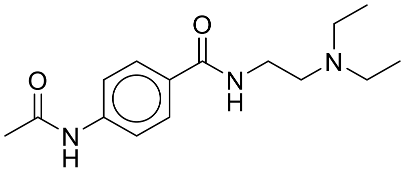	47.45
44	Pentoxifylline	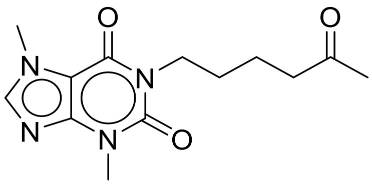	47.14
45	Prednisolone acetate	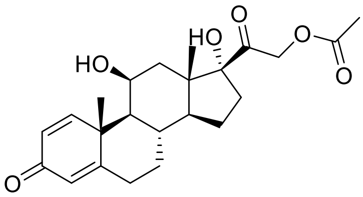	46.63
46	Palbociclib	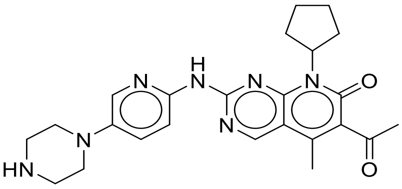	46.15

47	Moxisylyte hydrochloride	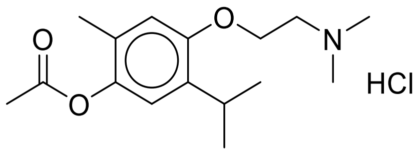	45.49
48	Aranidipine	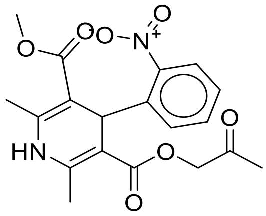	45.07
49	Benorilate	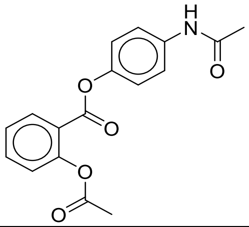	44.94
50	Testosterone acetate	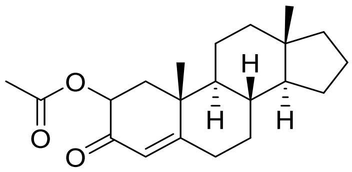	44.84
51	Retinyl acetate	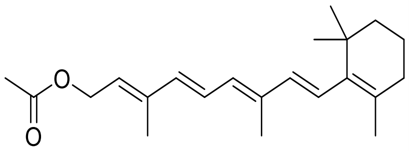	44.73
52	Acetohexamide	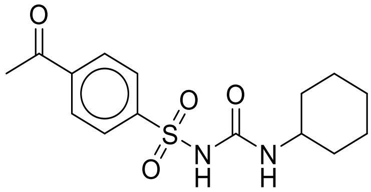	44.55
53	Acetylleucine	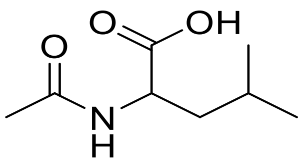	44.54
54	6-Acetamidohexanoic acid	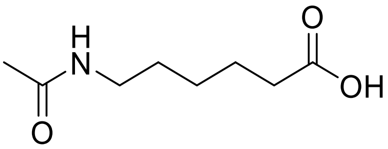	44.53

55	Nitazoxanide	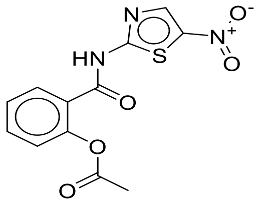	44.42
56	Celiprolol hydrochloride	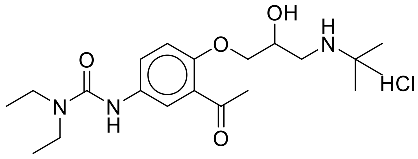	44.04
57	Propacetamol Hydrochloride	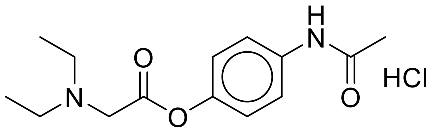	43.91
58	Actarit	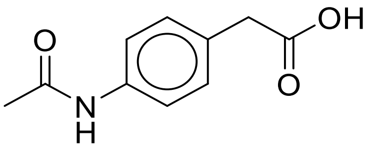	43.02
59	Acetyl-L-carnitine hydrochloride	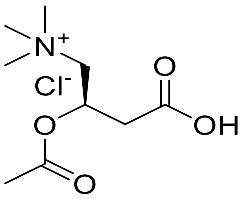	42.75
60	N-Acetyl-L-tyrosine	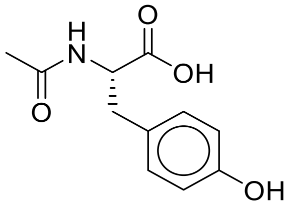	42.69
61	Sulfisoxazole acetyl	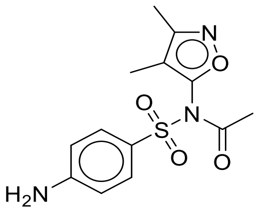	42.44
62	Acetophenazine dimaleate	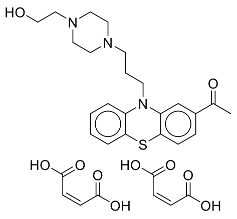	42.42

63	Nandrolone acetate	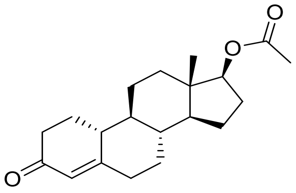	42.11
64	Triflusal	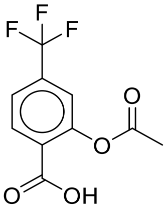	41.88
65	Nestoron	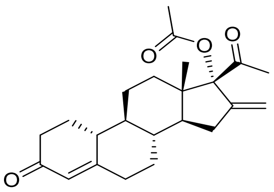	41.69
66	Spironolactone	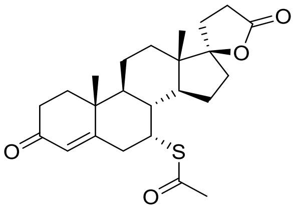	41.33
67	Melatonin	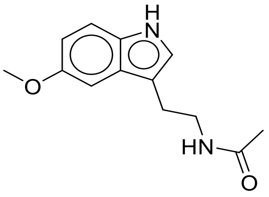	41.24
68	Triacetin	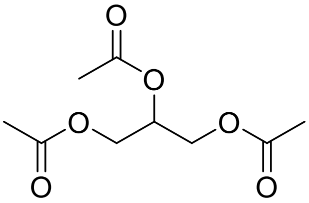	41.1
69	Aceglutamide	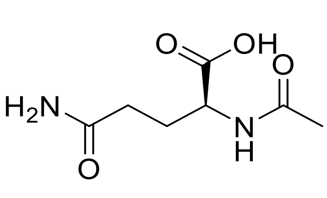	41.07
70	Dehydroisoandrostero ne 3-acetate	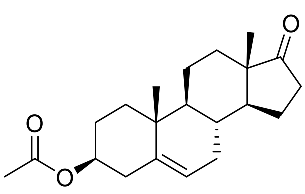	40.9

71	Vindoline	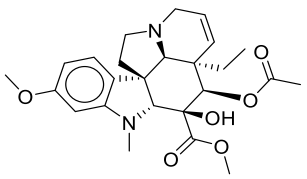	40.47
72	Norgestimate	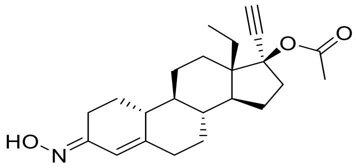	40.28
73	Ethopabate	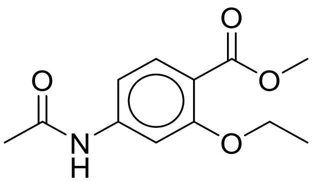	40.16
74	Progesterone	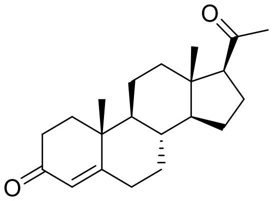	39.42
75	Oxaceprol	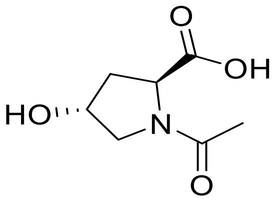	39.31
76	Agomelatine	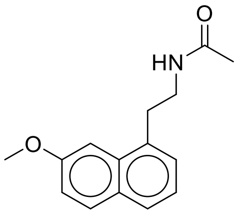	39.29
77	Nabumetone	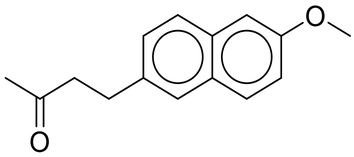	39.17
78	HMN-214	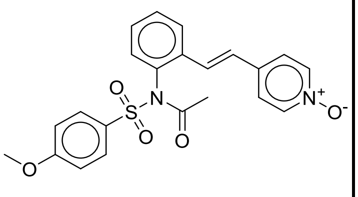	39.15

79	Eslicarbazepine Acetate	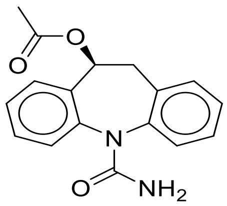	39.01
80	Aspirin	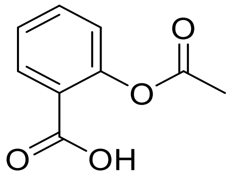	38.54
81	Acetylcysteine	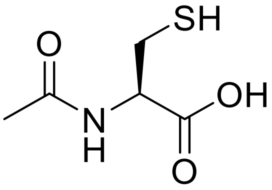	38.38
82	Vitamin K4	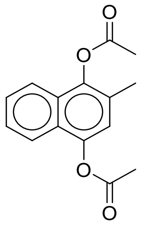	37.25
83	Dydrogesterone	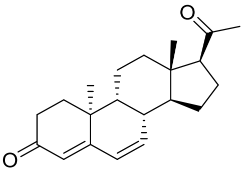	36.89
84	Pregnenolone	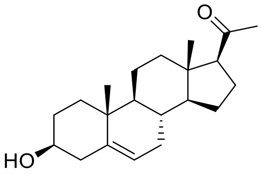	36.77
85	17α-Hydroxyprogesterone	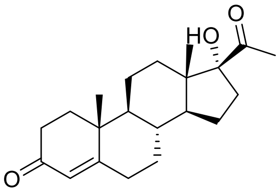	36.45
86	Medrysone	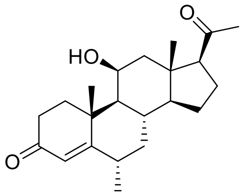	33.87

87	Phenacetin	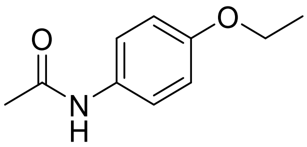	33.43
88	Methazolamide	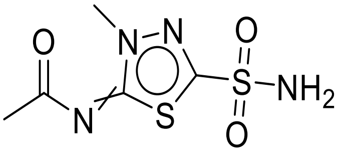	32.38
89	Bornyl acetate	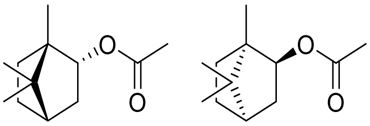	31.98
90	Sulfacetamide sodium	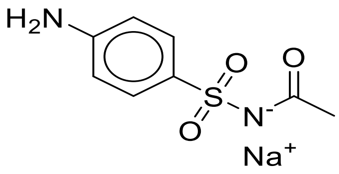	30.35
91	Methacholine Chloride	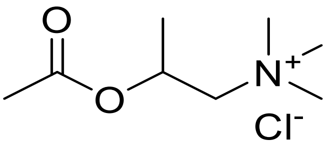	30.05
92	Paeonol	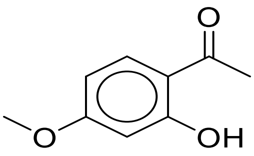	28.86
93	Dehydroacetic acid	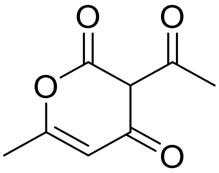	28.53
94	Citiolone	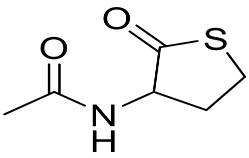	28.52

95	Fluorometholone	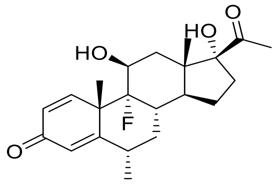	28.15
96	Acamprosate calcium	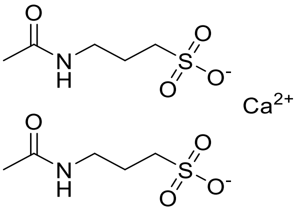	27.74
97	Resorcinol monoacetate	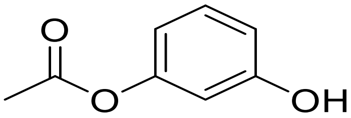	27.72
98	Acetylcholine chloride	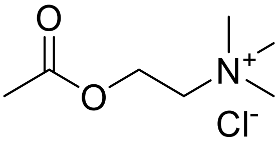	25.91
99	Ulipristal acetate	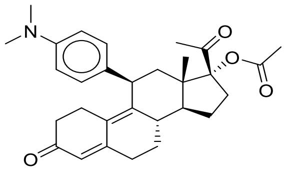	25.79
100	Acetaminophen	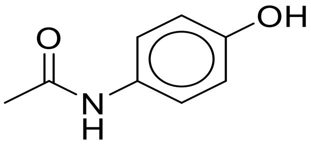	25.38
101	Acetylcholine bromide	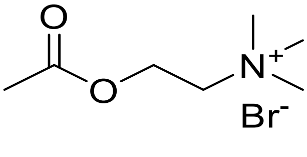	25.08
102	Acetylcholine iodide	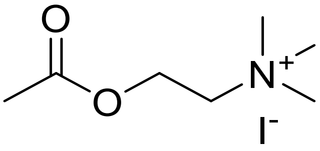	24.84

103	Medroxyprogesterone	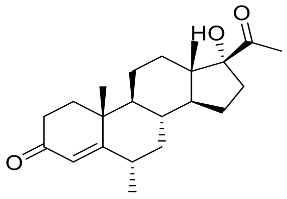	24.08
104	Nomegestrol acetate	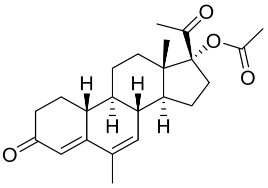	23.18
105	Acetohydroxamic acid	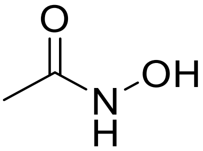	21.7
106	Deoxycorticosterone acetate	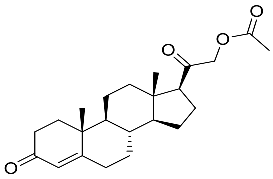	1.09

Supplementary table 2. The information of antibodies

antibody	supplier	catalogue number	dilution
Anti-acetylated-Lysine antibody	Cell Signaling Technology	9441s	1:1000
Anti-cGAS antibody (mouse)	Cell Signaling Technology	31659s	1:1000
Anti-TLR7 antibody	Cell Signaling Technology	82658s	1:1000
Anti-IRF3 antibody	Abcam	ab68481	1:1000
Anti-p-IRF3 antibody	Abcam	ab76493	1:1000
Anti-cGAS antibody	our laboratory	/	1:1000
Anti-Ac-cGAS (K384+K394) antibody	our laboratory	/	1:200
Anti-Ac-cGAS (K414) antibody	our laboratory	/	1:200
Anti-beta-actin antibody	Proteintech Group	66009-1-Ig	1:5000
Anti-GAPDH antibody	Proteintech Group	10494-1-AP	1:5000
Anti-alpha-tubulin antibody	Proteintech Group	66031-1-Ig	1:5000
Anti-TREX1 antibody	BD Biosciences	611986	1:1000
Goat Anti-Mouse IgM mu chain (Alexa Fluor 488)	Abcam	ab150121	1:500
Goat anti-Mouse IgG (H+L) Highly Cross-Adsorbed Secondary Antibody (Alexa Fluor 647)	Invitrogen	A21236	1:500
Anti-G3BP1 antibody	Proteintech Group	13057-2-AP	1:1000
PE anti-mouse CD11c antibody	Biolegend	117307	1:200
APC anti-mouse PDCA-1 antibody	Biolegend	127016	1:200

Supplementary table 3. The list of the sequences of qPCR primers.

Gene name	Forward primers	Reverse primers
Human *cGAS*	TAACCCTGGCTTTGGAATCAAAA	TGGGTACAAGGTAAAATGGCTTT
Human *TLR7*	CACATACCAGACATCTCCCCA	CCCAGTGGAATAGGTACACAGTT
Human *IFNB*	AGGACAGGATGAACTTTGAC	TGATAGACATTAGCCAGGAG
Human *GAPDH*	GAGTCAACGGATTTGGTCGT	TTGATTTTGGAGGGATCTCG
Mouse *Ifnb1*	TCCGAGCAGAGATCTTCAGGAA	TGCAACCACCACTCATTCTGAG
Mouse *Cxcl10*	GCCGTCATTTTCTGCCTCA	CGTCCTTGCGAGAGGGATC
Mouse *Ifit1*	GAACCCATTGGGGATGCACAACCT	CTTGTCCAGGTAGATCTGGGCTTCT
Mouse *Isg15*	TGACTGTGAGAGCAAGCAGC	CCCCAGCATCTTCACCTTTA
Mouse *Usp18*	TTGGGCTCCTGAGGAAACC	CGATGTTGTGTAAACCAACCAGA
Mouse *Oas1a*	GCCTGATCCCAGAATCTATGC	GAGCAACTCTAGGGCGTACTG
Mouse *Ccl2*	TTAAAAACCTGGATCGGAACCAA	GCATTAGCTTCAGATTTACGGGT
Mouse *Il6*	AGTTGCCTTCTTGGGACTGATG	GGGAGTGGTATCCTCTGTGAAGTCT
Mouse *Actb*	CAGGTCATCACTATTGGCAACGAGC	CGGATGTCAACGTCACACTTCATGA
Mouse *16s*	GTTACCCTAGGGATAACAGCGC	GATCCAACATCGAGGTCGTAAACC
Mouse *Nd4*	AACGGATCCACAGCCGTA	AGTCCTCGGGCCATGATT
Mouse *Dloop1*	AATCTACCATCCTCCGTGAAACC	TCAGTTTAGCTACCCCCAAGTTTAA
Mouse *Tert*	CTAGCTCATGTGTCAAGACCCTCTT	GCCAGCACGTTTCTCTCGTT
